# 60例原发乳腺弥漫大B细胞淋巴瘤的特征和生存分析

**DOI:** 10.3760/cma.j.cn121090-20251110-00519

**Published:** 2026-04

**Authors:** 焕倩 张, 嘉音 何, 思远 陈, 焕磊 张, 黎 王, 彭鹏 许, 维莅 赵

**Affiliations:** 1 上海交通大学医学院附属瑞金医院血液科，医学基因组学国家重点实验室，上海血液学研究所，上海 200025 Shanghai Institute of Hematology, State Key Laboratory of Medical Genomics, Department of Hematology, Ruijin Hospital, Shanghai Jiaotong University School of Medicine, Shanghai 200025, China; 2 山东第二医科大学附属益都中心医院血液科，潍坊 261000 Department of Hematology, Yidu Central Hospital Affiliated to Shandong Second Medical University, Weifang 261000, China; 3 山东第二医科大学附属益都中心医院科教科，潍坊 261000 Department of Education and Science, Yidu Central Hospital Affiliated to Shandong Second Medical University, Weifang 261000, China

**Keywords:** 淋巴瘤，大B细胞，弥漫性, 乳腺肿瘤, 基因突变, 预后, Lymphoma, large B-cell, diffuse, Breast neoplasms, Gene mutation, Prognosis

## Abstract

**目的:**

探究原发乳腺弥漫大B细胞淋巴瘤（PB-DLBCL）特征及预后相关因素。

**方法:**

回顾性收集上海交通大学医学院附属瑞金医院从2013年11月到2022年9月收治的60例PB-DLBCL患者的临床资料，并进行生存和预后因素分析。其中44例患者进行靶向测序（55个淋巴瘤相关基因）以评估基因突变情况及对生存结局的影响。

**结果:**

60例PB-DLBCL患者均为女性，中位年龄为56.5（49.0，61.0）岁，患者总体5年无进展生存（PFS）率和总生存（OS）率分别为66.5％（95％ *CI*：51.0％～86.7％）和90.6％（95％ *CI*：80.5％～100％）。一线治疗后完全缓解率为96.7％（58/60）。单因素分析结果显示LDH升高（*HR*＝3.380，95％ *CI*：1.160～9.791，*P*＝0.025）、非生发中心来源（non-GCB）型（*HR*＝3.560，95％ *CI*：1.000～12.700，*P*＝0.049）是PB-DLBCL患者PFS的不良预后因素，LDH升高是患者OS的不良预后因素（*HR*＝6.212，95％ *CI*：1.150～38.941，*P*＝0.034）。多因素分析未发现PB-DLBCL患者PFS的独立不良预后因素。靶向测序结果显示，PIM1、MYD88、KMT2D、CD79B、DTX1、MPEG1突变频率均高于20％。Log-rank检验分析显示PIM1突变患者（27例）的5年PFS率低于未突变患者（17例）［59.8％（95％ *CI*：39.7％～90.0％）对80.7％（95％ *CI*：58.3％～100％），*P*＝0.019］；MPEG1未突变患者（34例）的5年PFS率低于突变患者（10例）［59.4％（95％ *CI*：41.0％～86.1％）对100％，*P*＝0.048］；DTX1突变患者（10例）的5年OS率低于未突变患者（34例）［48.0％（95％ *CI*：18.8％～100％）对100％，*P*＝0.004］；MYD88突变患者（21例）的5年OS率低于未突变患者（23例）［75.4％（95％ *CI*：52.9％～100％）对100％，*P*＝0.004］。

**结论:**

PB-DLBCL患者5年OS率较高，PFS率较低。LDH升高、non-GCB型是影响患者生存的不良预后因素。PIM1、MYD88、KMT2D、CD79B、DTX1、MPEG1在PB-DLBCL中突变频率较高，PIM1、MYD88、DTX1突变的患者预后不佳，MPEG1突变的患者预后良好。

## Abstract (en)

**Objective:**

To investigate the characteristics and prognostic factors of primary breast diffuse large B-cell lymphoma（PB-DLBCL）.

**Methods:**

Clinical data on 60 patients with PB-DLBCL admitted to Ruijin Hospital, affiliated with Shanghai Jiao Tong University School of Medicine, from November 2013 to September 2022, were respectively collected, and survival and prognostic analyses were conducted. Among them, 44 patients underwent targeted sequencing（of 55 lymphoma-related genes）to assess the gene mutation status and its impact on survival outcomes.

**Results:**

All 60 patients with PB-DLBCL were female. The median age was 56.5 years（49.0, 61.0）. The overall 5-year progression-free survival（PFS）rate was 66.5％（95％ *CI*: 51.0％–86.7％）, and the 5-year overall survival（OS）rate was 90.6％（95％ *CI*: 80.5％–100.0％）. After one-line treatment, 96.7％（58 patients）achieved complete remission（CR）. The results of univariate analysis showed that elevated LDH（*HR*＝3.380, 95％ *CI*: 1.160–9.791, *P*＝0.025）and nongerminal center B-cell（non-GCB）type（*HR*＝3.560, 95％ *CI*: 1.000–12.700, *P*＝0.049) were adverse prognostic factors for PFS in patients with PB-DLBCL, whereas elevated LDH（*HR*＝6.212, 95％ *CI*: 1.150–38.941, *P*＝0.034）was an adverse prognostic factor for OS. Multivariate analysis did not identify independent adverse prognostic factors for PFS in patients with PB-DLBCL. Targeted sequencing results showed that the mutation frequencies of PIM1, MYD88, KMT2D, CD79B, DTX1, and MPEG1 were >20％. Log-rank test revealed that patients with PIM1 mutations（*n*＝27）had a significantly lower 5-year PFS rate compared to those without mutations（*n*＝17）［59.8％（95％ *CI*: 39.7％–90.0％）*vs* 80.7％（95％ *CI*: 58.3％–100％）, *P*＝0.019］. Regarding MPEG1, patients without mutations（*n*＝34）exhibited a significantly lower 5-year PFS rate than those with mutations（*n*＝10）［59.4％（95％ *CI*: 41.0％–86.1％）*vs* 100％, *P*＝0.048］. For DTX1, patients with mutations（*n*＝10）had a significantly lower 5-year OS rate than those without mutations（*n*＝34）［48.0％（95％ *CI*: 18.8％–100％）*vs* 100％, *P*＝0.004］. Similarly, patients with MYD88 mutations（*n*＝21）showed a significantly lower 5-year OS rate than those without mutations（*n*＝23）［75.4％（95％ *CI*: 52.9％–100％）*vs* 100％, *P*＝0.004］.

**Conclusion:**

The 5-year OS rate of PB-DLBCL is relatively high, whereas the PFS rate is relatively low. Elevated LDH levels and having the non-GCB type are adverse prognostic factors affecting patient survival. The mutation frequencies of PIM1, MYD88, KMT2D, CD79B, DTX1, and MPEG1 are higher in PB-DLBCL. Patients with mutations in PIM1, MYD88, and DTX1 have a poor prognosis, whereas those with MPEG1 mutations have a favorable prognosis.

2022年，全球非霍奇金淋巴瘤（NHL）新发人数达55.3万[1]，弥漫大B细胞淋巴瘤（DLBCL）占NHL的30％～40％。原发乳腺淋巴瘤诊断标准如下：淋巴瘤浸润灶与乳腺组织须紧密相关，既往无淋巴瘤病史或全身播散证据，允许存在同侧腋窝淋巴结受累[2]。一项多中心研究统计4 784例结外DLBCL患者显示，乳腺DLBCL患者占6.6％，其中符合原发乳腺DLBCL（PB-DLBCL）诊断标准的比例更低[3]。研究显示与累及胃肠道等其他结外部位的DLBCL相比，PB-DLBCL具有更强的侵袭性，预后更差[4]。主要治疗方式为免疫化疗（利妥昔单抗），其次为联合放疗和手术治疗，但后两者治疗选择存在争议。目前PB-DLBCL在临床实践中的精准管理仍面临挑战，主要源于对其临床异质性、分子遗传背景及预后决定因素的认识不足。本研究回顾性分析上海交通大学医学院附属瑞金医院的60例PB-DLBCL患者临床资料，旨在系统分析其临床特征、治疗、分子特征及生存结局方面的差异，以期为个体化治疗策略的制定提供依据。

## 病例与方法

1. 病例：本研究为回顾性队列研究，患者纳入标准：经系统性基线检查（含血常规、生化、心电图、骨髓穿刺及全身CT或PET-CT）；其次，病理诊断经由组织活检与免疫组化证实，并依据2016年WHO淋巴瘤分类标准复核；同时，CT或PET-CT影像学确认病灶累及乳腺，伴或不伴同侧腋窝或锁骨上淋巴结受累。排除标准：确诊时有广泛性淋巴瘤累及乳腺、既往有其他部位淋巴瘤病史、合并重大心脑肺肾功能障碍者、合并其他恶性肿瘤者均排除。基于以上标准，研究共纳入2013年11月至2022年9月期间于上海交通大学医学院附属瑞金医院初诊的60例PB-DLBCL患者作为研究对象。本研究已经上海交通大学医学院附属瑞金医院伦理委员会批准并豁免患者知情同意书［批件号：（2022）临伦审第（91）号］。

收集的患者一般资料与临床数据涵盖性别、年龄、美国东部肿瘤协作组（ECOG）评分、Hans分型、MYC/BCL2双表达（DE）状态、MYC基因和BCL2基因（少数为BCL6基因）的染色体易位或重排（DH）。单发或多发病灶、有无区域淋巴结转移，IPI评分、LDH（正常参考范围为120～250 IU/L）、全身增强CT或PET-CT影像报告、治疗方案、靶向测序结果，并随访其疾病转归及生存结局。

2. 治疗方案：本研究队列中，54例（90.0％）患者接受以R-CHOP（利妥昔单抗+环磷酰胺+阿霉素+长春新碱+泼尼松）为基础的化疗方案。其余6例（10.0％）患者接受了非R-CHOP方案治疗，主要包括DA-EPOCH-R（剂量调整的依托泊苷+泼尼松+长春新碱+环磷酰胺+阿霉素+利妥昔单抗）、布鲁顿酪氨酸激酶（BTK）抑制剂联合来那度胺与利妥昔单抗等。所有患者均在化疗后给予鞘内注射预防中枢神经系统（CNS）复发，具体方案（甲氨蝶呤10 mg+地塞米松5 mg+阿糖胞苷50 mg）。

3. 疗效评价及分期：本研究在化疗疗程结束后，采用PET-CT或全身多部位（颈部、胸部、腹部、盆腔）增强CT对治疗反应进行评估。疗效判定严格遵循2014版Lugano标准[5]，分为完全缓解（CR）、部分缓解（PR）、疾病稳定（SD）及疾病进展（PD）。患者的体能状态及预后风险分别采用ECOG评分及IPI评分进行评估。

4. 随访：本研究通过门诊复查、电话访谈及病历调阅进行随访，截止日期为2024年3月26日。无进展生存（PFS）期定义为从确诊日期至首次发生疾病进展、任何原因死亡或随访截止的时间。总生存（OS）期定义为从确诊至任何原因死亡或随访截止的时间。

5. 靶向测序基因突变：60例患者中，44例（73.3％）进行了靶向测序。本研究收集了55个淋巴瘤相关基因的检测数据，基因panel构成主要参考既往淋巴瘤分子分型研究[6]–[8]。应用组织基因组DNA（gDNA）提取试剂盒（Promega，美国，由上海源奇生物医药科技有限公司协助检测）提取gDNA，制备DNA全基因组文库。本研究基于Illumina NovaSeq测序平台进行测序。对获得的原始测序数据，借助CCDS、人基因组参考数据库（HG38）、dbSNP（v138）、1000 Genomes、COSMIC、PolyPhen-2及SIFT等多个权威数据库，以精准鉴定致病性的基因突变位点，致病性判定标准是Panel中的Ⅰ、Ⅱ、Ⅲ类突变（基于美国医学遗传学与基因组学学会联合分子病理学学会发布的2015指南）：Ⅰ类突变明确致病，可作为临床诊断依据；Ⅱ类突变极可能致病；Ⅲ类突变目前证据不足，无法判断致病与否。

6. 统计学处理：采用R语言4.5.1统计软件分析数据。定量资料以*M*（*Q*_1_,*Q*_3_）表示。所有分类变量均以频数（百分比）进行描述。采用Kaplan-Meier法绘制生存曲线，Log-rank检验比较组间PFS、OS的差异。预后因素分析首先进行单因素Cox回归，将其中*P*<0.05的变量进一步纳入多因素Cox比例风险模型进行独立预后因素分析，当稀疏数据限制了传统模型时，应用Firth校正（费尔斯惩罚似然）[9]。所有分析均以*P*<0.05为差异具有统计学意义。

## 结果

1. 患者临床特征分析：如表1所示，60例患者均为女性。患者总体中位年龄为56.5（49.0，61.0）岁。≥60岁16例（26.7％），LDH升高21例（35.0％），生发中心来源（GCB）型、DE及DH患者比例分别为46.7％、48.3％和0。乳腺多发病灶9例（15.0％），左侧乳腺受累29例（48.3％），双侧乳腺受累5例（8.3％）。区域淋巴结受累27例（45.0％），IPI评分≥ 2分7例（11.7％）（表1）。

**表1:** 60例原发乳腺弥漫大B细胞淋巴瘤患者临床特征［例（％）］

临床特征	统计值
年龄［岁，*M*（*Q*_1_，*Q*_3_）］	56.5（49.0，61.0）
年龄分层	
<60岁	44（73.3）
≥60岁	16（26.7）
性别	
女性	60（100）
男性	0（0）
细胞起源	
GCB型	28（46.7）
non-GCB型	32（53.3）
LDH水平	
升高	21（35.0）
正常	39（65.0）
DE	
是	29（48.3）
否	31（51.7）
DH	
是	0
否	60（100）
IPI评分（分）	
0	27（45.0）
1	26（43.3）
≥2	7（11.7）
ECOG评分（分）	
0	58（96.7）
1	2（3.3）
乳腺病灶	
单发	51（85.0）
多发	9（15.0）
受累乳腺位置	
左侧	29（48.3）
右侧	26（43.3）
双侧	5（8.3）
区域淋巴结（同侧腋窝/锁骨上）受累	
无	33（55.0）
有	27（45.0）
病灶手术切除	
完全切除	24（40.0）
只穿刺活检或部分切除	36（60.0）
放疗	
是	15（25.0）
否	45（75.0）

**注** GCB：生发中心来源；non-GCB：非生发中心来源；LDH：乳酸脱氢酶；DE：BCL2及MYC蛋白双表达；DH：MYC基因和BCL2基因（少数为BCL6基因）的染色体易位或重排；IPI：国际预后指数；ECOG：美国东部肿瘤协作组

2. 疗效评估与结局评价：60例患者均进行疗效评估，一线治疗后，58例CR、1例PD、1例SD。巨大包块（包块最大直径>5 cm）2例，1例诊断后15个月死亡。5例（8.3％）双侧乳腺受累患者均在一线治疗后达CR，其中1例诊断后71个月时出现进展，83个月死亡；1例诊断后58个月出现进展，61个月死亡；其余3例在随访期内无进展。截至末次随访PD 15例，死亡5例。死亡患者中3例（60％）发生CNS复发。患者中位随访时间41（95％ *CI*：33～52）个月，PB-DLBCL患者整体5年PFS率为66.5％（95％ *CI*：51.0％～86.7％），5年OS率为90.6％（95％ *CI*：80.5％～100％）。

亚组分析，LDH正常组（39例）5年PFS率［75.0％（95％ *CI*：59.5％～94.5％）对35.6％（95％ *CI*：8.6％～100％），*P*＝0.018］和OS率［94.1％（95％ *CI*：78.6％～100％）对90.5％（95％ *CI*：78.6％～100％），*P*＝0.021）均高于LDH升高组（21例）。GCB型组（28例）5年PFS率高于非GCB（non-GCB）型组（32例）［83.7％（95％ *CI*：63.5％～100％）对54.5％（95％ *CI*：35.6％～83.5％），*P*＝0.036］，而5年OS率差异无统计学意义（*P*>0.05）。

3. 单因素和多因素预后分析：单因素分析如表2所示，LDH升高（*HR*＝3.380，95％ *CI*：1.160～9.791，*P*＝0.025）、non-GCB型（*HR*＝3.560，95％ *CI*：1.000～12.700，*P*＝0.049）是患者PFS的不良预后因素。LDH升高是患者OS的不良预后因素（*HR*＝6.212，95％ *CI*：1.150～38.941，*P*＝0.034）。多因素分析结果未发现与PB-DLBCL患者PFS相关的独立不良预后因素（*P*值均>0.05）。

**表2:** 影响原发乳腺弥漫大Ｂ细胞淋巴瘤患者预后的单因素分析结果

影响因素	无进展生存		总生存^a^	
	*HR*（95％ *CI*）	*P*值	*HR*（95％ *CI*）	*P*值
年龄≥60岁	1.050（0.331～3.330）	0.935	3.362（0.532～21.491）	0.185
DE	0.744（0.252～2.200）	0.593	1.142（0.191～6.790）	0.877
LDH升高	3.380（1.160～9.791）	0.025	6.212（1.150～38.941）	0.034
多发病灶	1.940（0.596～6.293）	0.271	3.122（0.522～18.713）	0.196
non-GCB型	3.560（1.000～12.700）	0.049	2.417（0.446～24.157）	0.321
区域淋巴结受累	2.180（0.775～6.152）	0.139	0.839（0.141～4.987）	0.832
未手术切除	1.830（0.606～5.503）	0.285	0.660（0.108～4.030）	0.621
放疗	1.750（0.545～5.604）	0.348	0.648（0.025～16.692）	0.759

**注** DE：BCL2及MYC蛋白双表达；LDH：乳酸脱氢酶；non-GCB：非生发中心来源；^a^死亡事件仅有5例，应用Firth校正的Cox单因素分析，不进行多因素分析

4. 基因突变分析：44例进行靶向测序，55个淋巴瘤相关基因的突变情况如图1所示，其中PIM1（61％）、MYD88（48％）、KMT2D（32％）、CD79B（25％）、DTX1（23％）、MPEG1（23％）突变频率均高于20％。

**图1:**
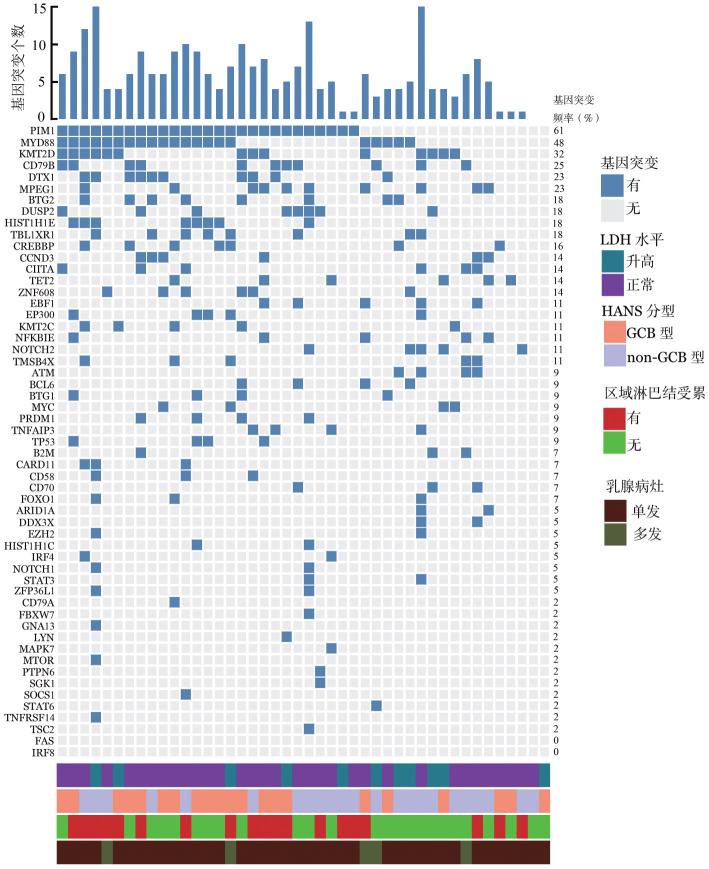
原发乳腺弥漫大B细胞淋巴瘤患者的基因突变特征 **注** GCB：生发中心来源；non-GCB：非生发中心来源；LDH：乳酸脱氢酶

将突变频率≥16％的11个基因进行生存分析，Log-rank检验结果显示：PIM1突变患者（27例）的5年PFS率低于未突变患者（17例）［59.8％（95％ *CI*：39.7％～90.0％）对80.7％（95％ *CI*：58.3％～100％），*P*＝0.019］；MPEG1未突变患者（34例）的5年PFS率低于突变患者（10例）［59.4％（95％ *CI*：41.0％～86.1％）对100％，*P*＝0.048］；DTX1突变患者（10例）的5年OS率低于未突变患者（34例）［48.0％（95％ *CI*：18.8％～100％）对100％，*P*＝0.004］；MYD88突变患者（21例）的5年OS率低于未突变患者（23例）［75.4％（95％ *CI*：52.9％～100％）对100％，*P*＝0.004］。其余7个基因的PFS与OS差异均无统计学意义（*P*值均>0.05）。

## 讨论

在结外淋巴瘤中，发生于乳腺的类型十分少见，研究报道原发乳腺淋巴瘤占结外NHL的比例不足2％[10]。组织学亚型以DLBCL最为常见[4]，可能由黏膜相关淋巴组织和炎症性淋巴结的刺激引起[11]。按照Wiseman的定义，根据有无区域淋巴结转移，PB-DLBCL分为ⅠE和ⅡE[12]，超声检查能够较早发现PB-DLBCL[13]。本病多见于中老年女性，男性发病比例不足4％[14]，本研究患者均为女性，中位年龄为56.5岁。

Weng等[14]研究结果显示PB-DLBCL患者5年PFS率为71.6％，5年OS率为84.8％。本研究PB-DLBCL患者5年PFS率为66.5％（95％ *CI*：51.0％～86.7％），5年OS率为90.6％（95％ *CI*：80.5％～100％），与上述研究相比PFS率略低而OS率略高。既往PB-DLBCL研究发现LDH水平与PFS和OS相关[15]–[16]，本研究中PB-DLBCL患者LDH升高是影响PFS和OS的不良预后因素，与既往研究一致。本队列中non-GCB型表现出了更差的PFS，也与朱悦红等[17]研究结论一致。既往研究发现区域淋巴结受累是预后不良因素[18]–[19]，而本队列中区域淋巴结受累非预后不良因素。据统计，DE在新诊断DLBCL病例中占20％～35％，并与不良预后相关[20]，本队列中DE患者占48％，较结内或其他结外淋巴瘤比例高，但非不良预后因素，乳腺多发病灶也非预后不良因素。因本队列双乳腺受累患者仅有5例（8％），ECOG评分1分以上的患者仅有2例（3％），样本量少，因此无法进行统计分析，本队列未发现DH。

目前，PB-DLBCL尚无统一的标准化治疗方案，临床治疗包括免疫化疗、放疗、鞘内注射化疗及联合手术治疗，但放疗、鞘内注射、手术存在争议。以利妥昔单抗为基础的免疫化疗，根据分子病理分型进一步选择RCHOP+X方案优于单纯RCHOP组[3]。本研究患者大多选择R-CHOP方案，疗效评估CR率达97％。本研究中化疗前手术已完全切除病灶的患者未改善PFS、OS，与既往研究[21]中乳腺切除术对提高OS并无明显优势的结果一致。在利妥昔单抗时代，有研究提出放疗对于PB-DLBCL仍具有显著的益处[22]，可降低乳腺复发风险[14]，但也有研究指出放疗虽可控制局部肿瘤复发，但并未提高PFS和OS率[15]。本研究放疗与未放疗组PFS和OS差异无统计学意义。Chan等[23]发现10例复发的PB-DLBCL患者中，有9例（90％）存在CNS受累情况。本研究死亡病例60％出现CNS复发，与之差异较大。对于CNS的预防仍存有争议，有研究显示接受预防性CNS治疗的患者预后优于未预防者[24]，接受高剂量甲氨蝶呤（HD-MTX）治疗的患者CNS受累风险低于未接受该治疗的患者[14]。但目前如无明确CNS浸润，临床很少在一线化疗方案中选择甲氨蝶呤。鞘内注射预防CNS复发值得推荐，本研究队列患者均进行CNS预防，这可能与我们有较低的CNS复发死亡率相关。但是也有报道显示预防性鞘内注射化疗不能降低CNS复发风险[23]。首次复发后行造血干细胞移植，可显著提高复发后的OS率和PFS率[23],[25]。本研究未发现与PB-DLBCL患者PFS相关的独立不良预后因素。

二代测序（NGS）解析疾病的分子突变特征，有助于阐明其分子机制及行分子分型。既往乳腺DLBCL患者NGS结果显示突变频率较高的基因包括PIM1、MYD88、CD79B、DTX1、KMT2D、PRDM1和CARD11[3],[14],[26]。本队列发现PIM1、MYD88、KMT2D、CD79B、DTX1、MPEG1突变频率均超过20％，与既往统计的高突变基因结果类似，MCD亚型为PB-DLBCL常见的分子亚型，这与Wright等[27]的研究一致，BTK抑制剂联合以利妥昔单抗为基础的免疫化疗有更好的治疗效果。PIM1突变、MPEG1未突变患者5年PFS率更低，尤其值得注意的是10例MPEG1突变的患者5年PFS率100％，显著高于未突变患者，提示野生型MPEG1是不良预后因素。DTX1突变、MYD88突变患者5年OS率低于未突变患者。

综上所述，PB-DLBCL作为一种特殊类型的结外DLBCL，LDH升高、non-GCB型是其不良预后因素，PIM1、MYD88、KMT2D、CD79B、DTX1、MPEG1是PB-DLBCL中常见的高突变基因。以上统计结果为单中心回顾性研究，样本量有限，部分亚组病例数少，统计分析结果存在一定局限性，未来尚需进一步扩大样本量进一步验证。

## References

[1] Lee HJ, Ramchandren R, Friedman J (2025). Brentuximab vedotin, nivolumab, doxorubicin, and dacarbazine for advanced-stage classical Hodgkin lymphoma[J]. Blood.

[2] Wiseman C, Liao KT (1972). Primary lymphoma of the breast[J]. Cancer.

[3] Chen SY, Xu PP, Feng R (2025). Extranodal diffuse large B-cell lymphoma: clinical and molecular insights with survival outcomes from the multicenter EXPECT study[J]. Cancer Commun (Lond).

[4] Cheah CY, Campbell BA, Seymour JF (2014). Primary breast lymphoma[J]. Cancer Treat Rev.

[5] Swain RS, Klink A, Asgarisabet P (2026). A novel real-world data methodology for lymphoma outcome classification: the real-world Lugano study[J]. J Comp Eff Res.

[6] Shen R, Fu D, Dong L (2023). Simplified algorithm for genetic subtyping in diffuse large B-cell lymphoma[J]. Signal Transduct Target Ther.

[7] Ramsower CA, Wright G, Li H (2025). Development and validation of a gene expression signature to predict early events in patients with follicular lymphoma[J]. Blood Adv.

[8] Li RC, Tang W, Zhang HL (2025). Clinical characteristics and molecular heterogeneity in follicular lymphoma with extranodal involvement[J]. Genome Med.

[9] Lee YJ, Kim M, Ahn SM (2025). Age-specific risk factors for cancer in a long-term Korean cohort of patients with ankylosing spondylitis treated with TNF inhibitors[J]. J Clin Med.

[10] Jia Y, Sun C, Liu Z (2018). Primary breast diffuse large B-cell lymphoma: a population-based study from 1975 to 2014[J]. Oncotarget.

[11] Arlen M, Freiman JJ, Ionescu M (2011). Infiltrating ductal carcinoma of the breast associated with primary breast lymphoma[J]. J Cancer.

[12] Franco F, González-Rincón J, Lavernia J (2017). Mutational profile of primary breast diffuse large B-cell lymphoma[J]. Oncotarget.

[13] Zhang XD, Zhang K (2023). Comparative analysis of conventional ultrasound and shear wave elastography features in primary breast diffuse large B-cell lymphoma[J]. World J Clin Cases.

[14] Weng H, Shrestha PR, Hong H (2023). Primary breast diffuse large B-cell lymphoma in the rituximab era: a retrospective study of the Chinese Southwest Oncology Group[J]. Cancer Med.

[15] Luo H, Yi P, Wang W (2019). Clinicopathological features, treatment, and prognosis in primary diffuse large B cell lymphoma of the breast: a retrospective study of 46 patients[J]. Med Sci Monit.

[16] Lu H, Luo L, Mi J (2025). Prognostic and clinicopathological role of soluble programmed cell death ligand-1 in patients with diffuse large B-cell lymphoma: a meta-analysis[J]. Front Oncol.

[17] 朱 悦红, 孟 文静, 何 丽宏 (2019). 原发性乳腺弥漫大B细胞淋巴瘤的预后分析[J]. 中华肿瘤杂志.

[18] Zhao P, Zhu L, Song Z (2020). Combination of baseline total metabolic tumor volume measured on FDG-PET/CT and β2-microglobulin have a robust predictive value in patients with primary breast lymphoma[J]. Hematol Oncol.

[19] Deng J, Mi L, Wang X (2022). Clinical prognostic risk analysis and progression factor exploration of primary breast lymphoma[J]. Hematology.

[20] Zhang M, Wu Y, Cheng Z (2025). Zanubrutinib plus R-CHOP improves the treatment effect of newly diagnosed diffuse large B cell lymphoma with double expression of MYC and BCL-2[J]. Front Immunol.

[21] Ryan G, Martinelli G, Kuper-Hommel M (2008). Primary diffuse large B-cell lymphoma of the breast: prognostic factors and outcomes of a study by the International Extranodal Lymphoma Study Group[J]. Ann Oncol.

[22] Liu PP, Wang KF, Jin JT (2018). Role of radiation therapy in primary breast diffuse large B-cell lymphoma in the rituximab era: a SEER database analysis[J]. Cancer Med.

[23] Chan CY, Ou CW, Chang H (2024). Primary breast diffuse large B-cell lymphoma characterized by CNS relapse and successful hematopoietic stem cell transplantation salvage therapy[J]. J Formos Med Assoc.

[24] 卫 锦杰, 王 列样, 赵 志强 (2024). 乳腺弥漫大B细胞淋巴瘤临床特征及预后分析[J]. 中国实验血液学杂志.

[25] Chen GL, Guo P, Wang J (2024). Predicting central nervous system relapse in primary breast diffuse large B-cell lymphoma using the stage-modified IPI score: A retrospective cohort study[J]. Heliyon.

[26] Zhang W, Huang C, Liu J (2022). Genomic mutation landscape of primary breast lymphoma: next-generation sequencing analysis[J]. Dis Markers.

[27] Wright GW, Huang DW, Phelan JD (2020). A probabilistic classification tool for genetic subtypes of diffuse large B cell lymphoma with therapeutic implications[J]. Cancer Cell.

